# Genetic rescue remains underused for aiding recovery of federally listed vertebrates in the United States

**DOI:** 10.1093/jhered/esad002

**Published:** 2023-03-28

**Authors:** Sarah W Fitzpatrick, Cinnamon Mittan-Moreau, Madison Miller, Jessica M Judson

**Affiliations:** W.K. Kellogg Biological Station, Michigan State University, Hickory Corners, MI, United States; Department of Integrative Biology, Michigan State University, East Lansing, MI, United States; Ecology, Evolution, and Behavior Program, Michigan State University, East Lansing, MI, United States; W.K. Kellogg Biological Station, Michigan State University, Hickory Corners, MI, United States; Ecology, Evolution, and Behavior Program, Michigan State University, East Lansing, MI, United States; Savannah River Ecology Lab, University of Georgia, Aiken, SC, United States; Division of Forestry and Natural Resources, West Virginia University, Morgantown, WV, United States; W.K. Kellogg Biological Station, Michigan State University, Hickory Corners, MI, United States; Ecology, Evolution, and Behavior Program, Michigan State University, East Lansing, MI, United States

**Keywords:** *assisted migration*, conservation, Endangered Species List, *gene flow*, translocation

## Abstract

Restoring *gene flow* among fragmented populations is discussed as a potentially powerful management strategy that could reduce inbreeding depression and cause *genetic rescue*. Yet, examples of *assisted migration* for *genetic rescue* remain sparse in conservation, prompting several outspoken calls for its increased use in genetic management of fragmented populations. We set out to evaluate the extent to which this strategy is underused and to determine how many imperiled species would realistically stand to benefit from *genetic rescue*, focusing on federally threatened or endangered vertebrate species in the United States. We developed a “*genetic rescue suitability index (GR index)*” based on concerns about small population problems relative to risks associated with *outbreeding depression* and surveyed the literature for 222 species. We found that two-thirds of these species were good candidates for consideration of *assisted migration* for the purpose of *genetic rescue* according to our suitability index. Good candidate species spanned all taxonomic groups and geographic regions, though species with more missing data tended to score lower on the suitability index. While we do not recommend a prescriptive interpretation of our GR index, we used it here to establish that *assisted migration* for *genetic rescue* is an underused strategy. For example, we found in total, “*genetic rescue*” was only mentioned in 11 recovery plans and has only been implemented in 3 of the species we surveyed. A potential way forward for implementation of this strategy is incorporating *genetic rescue* as a priority in USFWS recovery documentation. In general, our results suggest that although not appropriate for all imperiled species, many more species stand to benefit from a conservation strategy of *assisted migration* for *genetic rescue* than those for which it has previously been considered or implemented.

## Introduction

One of the central goals of conservation biology is to prevent population extirpations. Yet, population extirpations today are extremely common and are a main driver of the rapid defaunation of the planet (Ceballos *et al.* 2017). Many extirpations are caused by genetic and demographic factors associated with small population sizes, which can result in a positive feedback loop of compounding effects known as the “extinction vortex” (Gabriel and Bürger 1992; Frankham 2005). In this vortex, small populations experiencing strong genetic drift and inbreeding are subject to stochastic loss of genetic variation and increased frequency of deleterious alleles, both of which may reduce adaptive potential and absolute fitness (Bürger and Lynch 1995). Decreased absolute fitness leads to smaller population sizes, which accelerates these effects and further increases extinction risk due to demographic stochasticity (Lande 1993). Genetic threats to small populations have been empirically linked to population declines and extinctions in plants (Newman and Pilson 1997; Lennartsson 2002; Vilas *et al.* 2006), insects (Saccheri *et al.* 1998; Bijlsma *et al.* 2000; Wright *et al.* 2008), birds (Westemeier 1998), and mammals (Bozzuto *et al.* 2019).

The potential to ameliorate negative effects of genetic drift and inbreeding through *gene flow* is regularly discussed in the context of *genetic rescue* (e.g. Tallmon *et al.* 2004; Edmands 2006; Weeks *et al.* 2011; Frankham 2015; Bell *et al.* 2019). *Genetic rescue* is an increase in population fitness caused by the introduction of new genetic variation, i.e. *gene flow* (Tallmon *et al.* 2004; Whiteley *et al.* 2015). Research on *genetic rescue* has surged over the last decade, spanning theory (Robinson *et al.* 2021), empirical studies (Fitzpatrick *et al.* 2016; Hasselgren *et al.* 2018; Kronenberger *et al.* 2018; Robinson *et al.* 2021), and multiple reviews on the topic (Whiteley *et al.* 2015; Hedrick and Garcia-Dorado 2016; Bell *et al.* 2019; Ralls *et al.* 2020; Hoffmann *et al.* 2021). However, despite calls for its increased use as a conservation strategy (Frankham *et al.* 2017; Ralls *et al.* 2018), examples of *assisted migration* with the expressed purpose of *genetic rescue* remain sparse. A 2015 meta-analysis reported only ~20 cases globally of outcrossing or *assisted migration* among populations for purposes of *genetic rescue*, which the author notes is “probably a very low proportion of populations that would potentially benefit” (Frankham 2015). Since 2015 we found only 1 additional published example of *assisted migration* for the purpose of *genetic rescue* in an imperiled species (Weeks *et al.* 2017).

Importantly, when carried out, these efforts have almost always resulted in successful *genetic rescue*, evidenced by increased fitness following the onset of *gene flow* (Frankham 2015, 2016). Iconic examples of successful *genetic rescue* in vertebrates include Florida panthers (Johnson *et al.* 2010), greater prairie chickens (Westemeier 1998), bighorn sheep (Hogg *et al.* 2006), and mountain pygmy possums (Weeks *et al.* 2017). In all of these cases, increases in population abundance were observed following the onset of *assisted migration*, and presumably *gene flow*. Experimental studies have also shown consistent positive fitness effects of *gene flow*, especially into small populations, under a range of experimental conditions and taxonomic groups (Hufbauer *et al.* 2015; Kronenberger *et al.* 2017, 2018; Robinson *et al.* 2017). Altogether, Frankham’s (2015) meta-analysis of 156 relevant datasets, including many controlled experiments as well as field studies, found that outcrossing or *assisted migration* among populations was beneficial to inbred populations in 93% of cases, and that *genetic rescue* effects persisted to at least the F3 generation (Frankham 2016). Given the potential boon this strategy may offer for recovery of small populations, understanding why *assisted migration* is used so infrequently and providing conservation practitioners clear guidelines for implementation are urgent priorities.

Recently, multiple independent groups with global expertise in evolutionary conservation biology have called for increased attention to *gene flow* restoration and *genetic rescue* in conservation and management (Hedrick and Garcia-Dorado 2016; Bell *et al.* 2019; Kardos *et al.* 2021; Willi *et al.* 2022). One of the most outspoken messages was a “call for a paradigm shift in the genetic management of fragmented populations” (Ralls *et al.* 2018); a message that was reiterated in a textbook written specifically for conservation practitioners (Frankham *et al.* 2017). The central argument is—for organisms with low risk of *outbreeding depression*, the default management strategy should shift from maintaining separation among distinct lineages to a new default of evaluating whether restoring *gene flow* among populations is necessary, appropriate, and feasible. This proposed shift favors a proactive over a “do-nothing” approach to the management of fragmented populations. While frameworks for making evidenced-based genetic management decisions exist (i.e. Frankham *et al.* 2011; Ralls *et al.* 2018), there have not been any specific evaluations of how many and which protected species stand to benefit from *assisted migration* and *genetic rescue*.

Despite many potential unrealized benefits, *genetic rescue* is by no means a panacea for all endangered or threatened species. An important initial step is determining the extent to which a species is a viable candidate for *genetic rescue*. Species that will benefit most from restored *gene flow* are those with small, isolated populations that have been recently fragmented by human activities (Frankham *et al.* 2011, 2017). Recently fragmented populations (i.e. fragmentation associated with human-induced land use change within the past 200 yr) are most vulnerable to the fitness consequences of expressed *genetic load* associated with genetic drift and inbreeding (Bertorelle *et al.* 2022). On the other hand, there are several characteristics that increase the probability of *outbreeding depression* and thus would deem a species a poor candidate for *assisted migration* with the purpose of *genetic rescue*. For example, populations with deep divergence histories, fixed chromosomal differences, and/or adaptive differentiation are generally at higher risk of *outbreeding depression* (Frankham *et al.* 2011). Developing general guidelines for predicting the probability of *outbreeding depression* is challenging due to differences in natural history, evolutionary and demographic history, and the environmental context. This uncertainty is often discussed as a deterrent to *assisted migration* (Edmands 2006). However, it is increasingly recognized that *outbreeding depression* can be largely predictable given sufficient natural history and genetic information for a species, and rules of thumb are provided in a recent “practical guide” written specifically for fish and wildlife managers (Frankham *et al.* 2019).

Our study was motivated by 2 premises: 1) the increasing calls for wider use of *assisted migration* for *genetic rescue* in conservation and management, and 2) the factors underlying good versus risky candidates for such a strategy are generally well understood. Yet, surveys of how often *assisted migration* for *genetic rescue* is actually considered or implemented seem to be lacking and we do not know the extent to which this strategy is truly underused. Our goal in this study was to address these unknowns by surveying federally threatened or endangered vertebrate species on the US Endangered Species List. We used a common set of criteria to develop a “*genetic rescue* suitability index” to evaluate the potential suitability of each species for *assisted migration* and *genetic rescue*. We were also interested in identifying trends among taxonomic groups or geographic regions that have disproportionately high or low numbers of good candidate species for *genetic rescue*. Ultimately, we aim to contribute to the path forward for implementation of the “paradigm shift in genetic management of fragmented populations.”

## Methods

### Filtering species survey list

To understand the extent to which *assisted migration* for *genetic rescue* is an underused conservation strategy, we first generated a list of species to evaluate. We limited our evaluation to federally listed vertebrate species in the United States, though we note that *genetic rescue* is likely also beneficial for many non-US species and for other taxonomic groups. We used the U.S. Fish & Wildlife Service (FWS) Environmental Conservation Online System (ECOS) to query all vertebrate species listed by the FWS. On 1/20/2022, we downloaded this list as a.csv file (https://ecos.fws.gov/ecp/report/species-listings-by-tax-group?statusCategory=Listed&groupName=All%20Vertebrate%20Animals&total=415). We filtered the “Endangered Species Act Listing Status” column for species that were Endangered or Threatened, excluding categories of “Experimental Population, Non-essential” and “Similarity of Appearance.” We followed the Endangered Species Act’s definition of a species, as including “any subspecies…and any distinct population segment of any species of vertebrate fish or wildlife which interbreeds when mature.” Some species had multiple records due to multiple *Distinct Population Segment* (DPS) listings. For the purposes of our survey, we collapsed these into a single record per species and noted locations where each was listed. For example, most species are listed “Wherever found,” but some only have specific DPS listed. We recorded scientific and common name, the FWS Lead Region, whether the species or DPS was Endangered or Threatened, and taxonomic group. Next, given that we relied on NatureServe information for our survey (described below), we filtered out species that were not found on NatureServe, which mostly included species from US territories such as Guam or Caribbean species. We excluded species or subspecies with fewer than 2 populations and species that are likely extinct according to recent surveys, as *assisted migration* for *genetic rescue* is not possible for these species. Additionally, we removed all Hawaiian and other island species, as these species were often represented by a single island. We further excluded entirely marine species such as sea otters and sea turtles because of either the existence of single population units, and thus no opportunity for *genetic rescue*, or the difficulty that would be presented by not having a specific regional target for *assisted migration*. Finally, we removed the Thick-billed Parrot, as there are no remaining populations in the United States. These filtering steps resulted in a final list of 222 vertebrate species or subspecies across 8 U.S. FWS Lead Regions in the contiguous United States and Alaska.

The species list was divided evenly across 3 authors (CM-M, JMJ, and SWF). In addition to the information listed above, we recorded the year of listing on the Endangered Species List, and the year of most recent species status documentation for each species or subspecies, which included *Recovery Plans* (RP), *Species Status Assessments* (SSA), and *5-year reviews* associated with the FWS species listing—henceforth referred to as “recovery documentation.” We used NatureServe Explorer’s “Estimated Number of Element Occurrences” information (https://explorer.natureserve.org/) to approximate the remaining number of populations for each species. We further recorded basic habitat information from NatureServe Explorer and age at maturity and lifespan (in years) according to information from both NatureServe and ECOS. If we could not find information from these 2 sources, we used the Advanced search feature of Google Scholar (https://scholar.google.com/) using the species’ scientific name and “age at maturity” or “lifespan” to find relevant literature. Finally, we recorded the FWS “*Recovery Priority number*” for each species. The *Recovery Priority number* is based on the species’ or populations’ Recovery Potential (e.g. degree of threat, taxonomic uniqueness, probability of recovery, Supplementary Table S1) and Taxonomy (Table 2), and ranges from 1 (highest) to 18 (lowest) priority. These scores fall into 3 broad priority categories, High, Moderate, and Low (Table 2).

### Evaluation of *genetic rescue* suitability index

Our goal was to generate a “*genetic rescue suitability index*” (GR index) reflective of the suitability of *assisted migration* with the purpose of *genetic rescue* for each listed species or subspecies. In general, successful *genetic rescue* following *assisted migration* requires that the species have multiple, distinct populations that can be connected via *gene flow*. These populations must differ in their genetic variation, but would ideally occupy similar habitats or ecoregions, exhibit similar chromosomal structure, and lack barriers to reproductive success, such as differences in mating behaviors, or mating phenology (Frankham *et al.* 2011). Populations that stand to benefit the most from augmented *gene flow* are those experiencing decreased fitness due to low genetic variation, inbreeding depression, and/or small population sizes (Whiteley *et al.* 2015). Lastly, as with any conservation strategy, the more information known about the species (i.e. information about disease, behavior, genetic variation), the more tailored and successful conservation actions are likely to be. With these considerations in mind, we surveyed the literature to answer a set of questions designed to evaluate the potential success and risks of *genetic rescue* in each threatened or endangered species.

We searched the species name on NatureServe Explorer and, if necessary, found subspecies information related to the listing. We then downloaded all FWS recovery documentation from ECOS. Finally, we performed an “Advanced search” on Google Scholar with the following parameters: containing the exact phrase of the species’ scientific name and containing at least one of the words “genetic,” “inbreeding,” and/or “translocation.” All relevant literature was downloaded for use in answering scoring questions. Each species was assigned a unique ID associated with a folder that contained all literature and FWS documentation used for scoring.

The GR index was the sum of scores from 9 questions about the species or subspecies, with NAs treated as zeros (Table 1). Species with higher GR indices are better candidates for considering *assisted migration* for *genetic rescue* than those with lower scores. Questions 1 to 4 assessed concerns about classic “small population problems.” Inbreeding depression, low genetic variation, and small population sizes are related, yet distinct conditions that can occur simultaneously or independently in isolated populations. For example, a population may have low genetic variation but show no apparent signs of inbreeding depression, either because the population is not inbred, or because inbreeding depression is often expressed in an environment-specific context (Keller and Waller 2002). Alternatively, a population could have small census size and be vulnerable to demographic stochasticity without necessarily having lost much genetic variation. Thus, we opted to score these questions separately under the rationale that if a species shows evidence of all 3 (i.e. inbreeding depression, low genetic variation, and small population size), it should be weighted as a stronger candidate for *genetic rescue* than a population only showing evidence of one of these.

**Table 1.** Questions and possible responses used in species surveys for assigning a GR index.

Question	Possible responses
1.Are there concerns or signs of inbreeding depression?	No evidence is specifically stated = 0 Concerns of inbreeding depression = 1 Signs of inbreeding depression = 2 Inbreeding concerns not mentioned = NA
2.Are there concerns about low levels of genetic variation?	No concern is stated = 0 Yes = 1 No information about genetic variation = NA
3.Are there concerns about small population sizes?	No concern is stated = 0 Yes = 1 Population size not mentioned = NA
4.Have population genetic surveys been performed?	No = 0 Yes = 1
5.What is the ploidy of the species?	Diploid = 0 Polyploid = −1
6.Is the only option of genetic rescue to reconnect populations that have known adaptive differentiation?	No concern is stated, or adaptive variation similar across multiple populations = 0 Yes = −1 Unknown or not mentioned = NA
7.Is the only option to reconnect populations from distinct ecoregions?	No = 0 Yes = −1
8.Are there disease concerns associated with crossing populations?	No concern is stated = 0 Yes = −1 Disease is not mentioned or is unknown = NA
9.Are there population-specific behaviors that could complicate translocations among populations?	Yes = −1 No behavioral concerns mentioned = NA

*Q1. Are there concerns or signs of inbreeding depression?*

Evidence of inbreeding depression is one of the clearest of all signals that a population would benefit from *assisted migration*. Species were scored as follows: Species where fitness or phenotypic effects of inbreeding were explicitly mentioned, +2; inbreeding depression was stated to be an explicit concern but no concrete evidence was presented, +1; absence of inbreeding depression explicitly mentioned, 0; no mention of inbreeding depression at all, “NA.”

*Q2. Are there concerns about low levels of genetic variation?*

Even without clear signs of inbreeding depression, levels of genetic variation can offer insight into the prevalence of inbreeding and adaptive potential in the population (Kardos *et al.* 2021). Species were scored as follows: some level of concern about low genetic variation mentioned, +1; explicit statement that low genetic variation is not a concern, 0; no mention of genetic variation, “NA.”

*Q3. Are there concerns about small population sizes?*

Habitat fragmentation not only reduces *gene flow* among isolated populations, but it often reduces habitat available to support the past carrying capacities of restricted species (Didham *et al.* 2012). Small populations are more vulnerable to inbreeding than large populations, and when small population size is coupled with low genetic variation and inbreeding, adaptive capacity is reduced (Frankham *et al.* 1999; Skelly *et al.* 2007; Bijlsma and Loeschcke 2011; Klerks *et al.* 2019). Further, small population sizes are inherently more vulnerable to extinction caused by demographic stochasticity (e.g. Lande 1993). Species were scored as follows: concerns of small population sizes stated in the literature, +1; explicit statement that small population size is not a concern, 0; population size was not mentioned, “NA.”

*Q4. Have population genetic studies been performed?*

Population genetic studies are invaluable for understanding inbreeding and genetic variation in endangered populations and for identifying distinct population units that could be adapted to different environmental conditions (Allendorf *et al.* 2010; Kardos *et al.* 2021). As these studies can directly inform the applicability of *genetic rescue*, we searched the literature (see above) for published population genetic information, including dissertations and recovery documentation. We limited studies to those with population-level sampling, excluding taxonomic studies based on one or a small number of individuals per species. Species were scored as follows: genetic studies present, +1; no population genetic information found, “NA.” We also recorded the genetic marker used for these studies.

Questions 5 to 9 addressed potential risks associated with *assisted migration* for *genetic rescue*, including risk factors for *outbreeding depression* and species characteristics that could hinder *assisted migration* efforts.

*Q5. What is the ploidy of the species?*

Polyploidy is a common concern for *genetic rescue*, as matings between individuals of different ploidy levels are unlikely to result in viable offspring (Schmidt-Lebuhn *et al.* 2018). While intraspecies variation in ploidy number is rare in vertebrates, some fish families exhibit variation (e.g. Acipinseridae, Catostomidae, Salmonidae; Comber and Smith 2004). We assessed whether ploidy differed across populations by searching the materials for evidence of polyploidy. If no variation in ploidy was documented for the species, we assumed it was diploid. Species were scored as follows: variation in ploidy between populations, −1; no variation (i.e. all diploid), 0.

*Q6. Is the only option of genetic rescue to reconnect populations that have known adaptive differentiation?*

We searched all literature for mentions of adaptive phenotypic variation across populations of the species. Species were scored as follows: trait(s) differed across populations, and the only option for *assisted migration* would be to cross populations with known adaptive differences, −1; explicit reference to a lack of interpopulation differences, 0; adaptive differentiation not mentioned, “NA.” When species were scored as −1, we listed the specific traits which differed.

*Q7. Is the only option to reconnect populations from distinct ecoregions?*

Similarly, we searched all literature to identify the locality of each remaining population and compared them to the EPA Level I EcoRegions map (https://gaftp.epa.gov/EPADataCommons/ORD/Ecoregions/cec_na/NA_LEVEL_I.pdf). Species were scored as follows: each population is found in a different ecoregion suggestive of differences in the environments experienced by the populations under investigation, −1; species had at least 2 populations in the same ecoregion, 0.

*Q8. Are there disease concerns associated with crossing populations?*

Introducing a novel disease would be detrimental to conservation efforts, and disease risk has long been a concern of translocation efforts worldwide (Cunningham 1996; Berger-Tal *et al.* 2020). Species were scored as follows: disease, which we considered to include all infectious agents (e.g. viruses, bacteria, fungi, ectoparasites), mentioned as a concern in the literature for the species, −1; no disease concerns are currently warranted, 0; disease concerns not mentioned or unknown, “NA.” When disease was listed as a concern, we recorded the specific pathogen of concern.

*Q9. Are there population-specific behaviors that could complicate translocations among populations?*

Behavioral differences among populations that might hinder successful *assisted migration* and *genetic rescue* could include differences in reproductive behaviors and phenology, homing behavior, and foraging behavior (reviewed in Berger-Tal *et al.* 2020). We searched the collected literature for mention of behaviors that differ between populations (excluding differences between captive-raised and wild-born individuals). Species were scored as follows: behavioral differences among populations mentioned, −1; behavioral differences not mentioned, “NA.” We recorded the specific behavioral differences where present.

Finally, for each species we noted whether translocations, which we define here as any form of human-assisted movement of animals from one location to another, had been considered, implemented, or not mentioned/performed. We included translocations where individuals from captive breeding programs or hatcheries were released into the wild, translocations from a wild population to previously inhabited areas, and translocations between populations. We also searched all recovery documentation for the term “*genetic rescue*” and indicated whether *genetic rescue* was mentioned. If a plan discussed the general concept of *genetic rescue* (i.e. human-*assisted migration* for restoring *gene flow* and increasing population growth) we noted this as well.

### Statistical analysis

All statistical analyses were performed in R v 4.0.2 (R Core Team 2022). We first tested for differences in GR index distributions among the 3 evaluators. GR index was not normally distributed and had many tied values. To account for this, we used the Kruskal–Wallis test, a nonparametric test for comparing whether 2 or more groups of samples come from the same distribution. The Kruskal–Wallis test statistic, *H*, confirmed that the distribution of GR index did not differ among evaluators (*H* = 1.89; *P* = 0.39). Thus, we combined all GR indices across authors for analysis. We used the Kruskal–Wallis test to understand differences in the distribution of GR index across taxonomic groups, U.S. FWS Lead Region, number of populations remaining according to NatureServe, and translocation status (implemented, considered, or not mentioned/performed). When *H* was significant, we used Dunn’s test to determine which specific categories differed in GR index distribution. Dunn’s test was performed with Bonferroni adjustment in the “dunn.test” R package v 1.3.5, which accounts for tied ranks (Dinno 2017). We used a Mann–Whitney *U* test to determine whether the distribution of GR index differed between species listed as threatened and those listed as endangered and to assess the relationship between GR index and age at maturity. Age at maturity was split into 2 categories: species that matured before the age of 4 yr were classified as “fast life-history” and those that matured at 4 yr or older were classified as “slow life-history.” To test whether there was a relationship between translocation status and taxonomic group, we used Fisher’s exact test. We assessed the correlation between GR index and the number of survey questions answered “NA” and the correlation between GR index and Recovery Priority category (High, Moderate, or Low, Table 2) with Kendall’s rank correlation coefficient (Kendall’s *τ*). As the number of remaining populations was reported as a range of values (e.g. “1 to 5,” “6 to 20”), we analyzed the relationship between GR index and number of remaining populations in 2 ways: we treated number of remaining populations as a categorical variable with the Kruskal–Wallis test, and we also converted the population value ranges to an ordinal variable and assessed Kendall’s rank correlation coefficient. Similarly, we treated the number of remaining populations as an ordinal variable and used a Kruskal–Wallis test to assess the relationship between translocation status and number of populations remaining. We used “ggplot2” v 3.3.5 (Wickham 2016), “png” v 0.1-7 (Urbanek 2013), and “patchwork” v 1.1.1 (Pedersen 2020) for plotting figures.

**Table 2.** U.S. Fish and Wildlife Recovery Priority guidelines.

Prioritization category	Recovery Potential	Taxonomy	Priority Number
High	High	Monotypic genus	1
		Species	2
		Subspecies/DPS	3
	Low	Monotypic genus	4
		Species	5
		Subspecies/DPS	6
Moderate	High	Monotypic genus	7
		Species	8
		Subspecies/DPS	9
	Low	Monotypic genus	10
		Species	11
		Subspecies/DPS	12
Low	High	Monotypic genus	13
		Species	14
		Subspecies/DPS	15
	Low	Monotypic genus	16
		Species	17
		Subspecies/DPS	18

Adapted from Federal Register Document 83-25716, 1983. Lower numbers indicate species with higher priority.

## Results and discussion

**Fig. 1.**
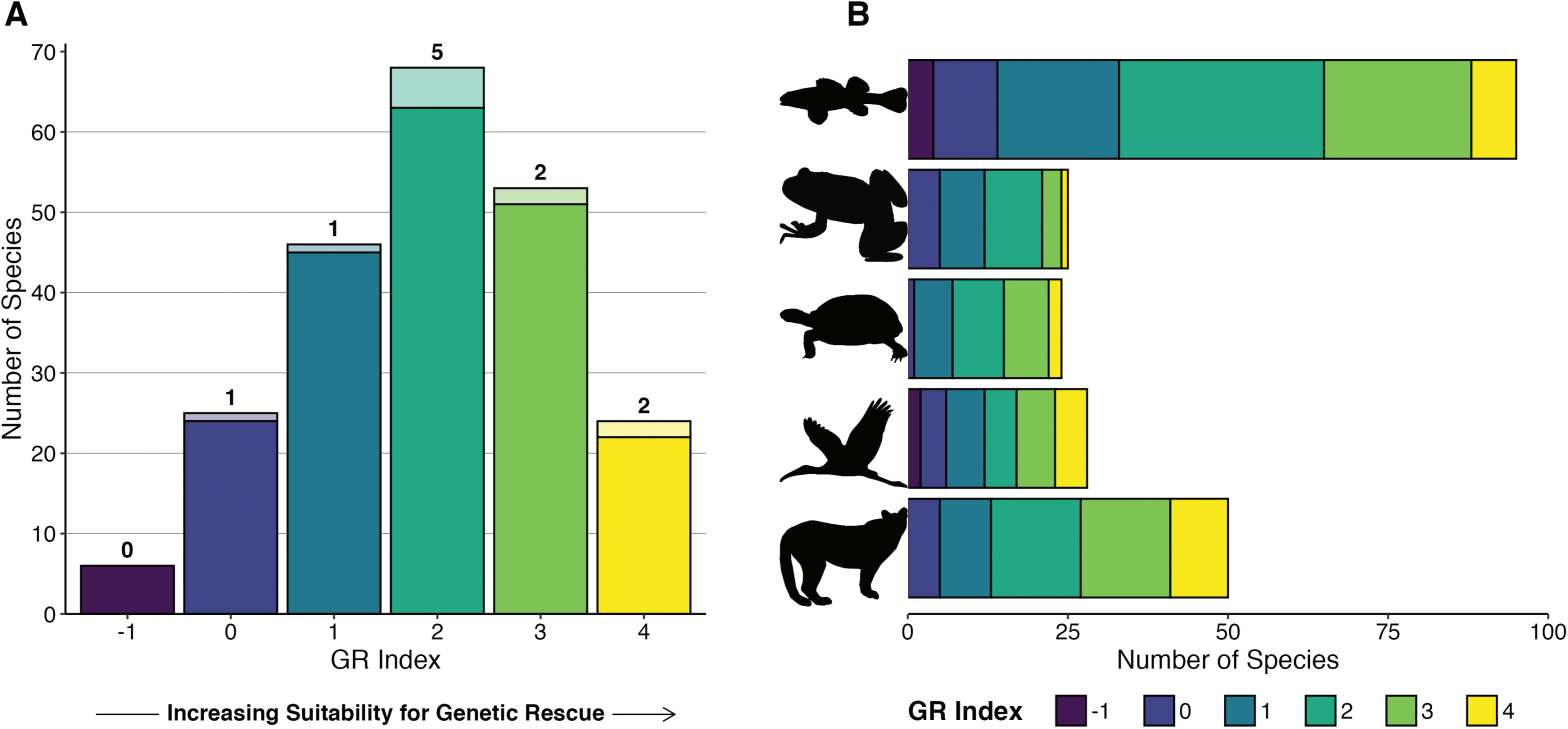
Distribution of GR index across endangered and threatened vertebrates listed by the U.S. Fish & Wildlife Service. A) GR index across all vertebrates surveyed. Numbers and lighter shading represent species that had explicit mention of genetic rescue in recovery documents. B) GR index across taxonomic groups. Silhouettes were downloaded from PhyloPic; desert tortoise silhouette ©Andrew A. Farke (https://creativecommons.org/licenses/by/3.0/legalcode).

Our overarching goal was to evaluate the sentiment that *assisted migration* and *genetic rescue* are underused strategies in the genetic management of fragmented populations, and to provide some suggested paths forward. We did this by developing a “*genetic rescue* suitability index” (GR index) with which to survey imperiled species. Across the 222 evaluated species, GR index ranged from negative 1 to 4 (Fig. 1A). A third of species were assigned a GR index of 2 and 65% of species were assigned a GR index of 2 or higher. Species for which *assisted migration* for the purpose of *genetic rescue* has already occurred (i.e. Columbia Basin Pygmy Rabbit, Florida Scrub-jay, and Florida Panther) received high GR indices (≥3), providing confidence that our scoring system did identify known candidate species for this strategy (Table 3). Our GR index also appropriately identified species that intuitively would not be good candidates for *assisted migration* for *genetic rescue* and thus had low GR indices, such as polyploids, species with behavioral or disease concerns, and species with excessive missing data. We interpreted species with a GR index of 2 or higher as worthwhile candidates for consideration of *assisted migration* for *genetic rescue*. Consideration for this strategy does not necessarily mean that *assisted migration* will ultimately be appropriate for all high-scoring species following full evaluation of potential risks and practicality. However, our evaluation suggests nearly two-thirds of listed vertebrate species should be considered compared with <5% of species for which *genetic rescue* was mentioned in the recovery documentation. Once a species is deemed a strong candidate for *assisted migration* for *genetic rescue*, and logistical barriers are overcome, the implementation of this strategy is likely to produce beneficial outcomes.

**Table 3.** Species accounts in which “genetic rescue” was mentioned in USFWS Recovery documentation.

Scientific name	Common name	Lead Region	ESA listing status	Taxonomic group	GR index	GR status	Assisted migration scenario(s)	Outcome
*Aphelocoma coerulescens*	Florida scrub-jay	Southeast	Threatened	Birds	4	Implemented	(1) ~50 Florida scrub-jays translocated from sites within the same metapopulation to an existing population of ~15 (2) Eight jays translocated from a large central FL population into a small and isolated population of ~75 on the Atlantic coast	(1) Population expansion but decreased heterozygosity due to high success of a few immigrant jays (Fitzpatrick pers. comm) (2) Ongoing, TBD
*Brachylagus idahoensis*	Columbia Basin Pygmy Rabbit	Pacific	Endangered	Mammals	4	Implemented	Columbia Basin Pygmy Rabbits crossed with Idaho Pygmy Rabbits in captivity then released to the wild along with Pygmy rabbits from 4 other states	Genetic rescue in captivity (increases in vital rates; Elias *et al.* 2013); population expansion in the wild, but intensive management needed due to wildfires and high levels of predation (Nerkowski 2021)
*Puma (=Felis) concolor coryi*	Florida panther	Southeast	Endangered	Mammals	3	Implemented	Eight female panthers from a different subspecies introduced to resident FL population of 34 inbred individuals	Genetic rescue; increases in population size, heterozygosity, decreased signs of inbreeding (Johnson *et al.* 2010)
*Ambystoma bishopi*	Reticulated flatwoods salamander	Southeast	Endangered	Amphibians	2	Considered	N/A	N/A
*Drymarchon corais couperi*	Eastern indigo snake	Southeast	Threatened	Reptiles	0	Considered	N/A	N/A
*Eremophila alpestris strigata*	Streaked Horned lark	Pacific	Threatened	Birds	2	Considered	N/A	N/A
*Oryzomys palustris natator*	Silver rice rat	Southeast	Endangered	Mammals	3	Considered	N/A	N/A
*Oncorhynchus clarkii seleniris*	Paiute cutthroat trout	Pacific Southwest	Threatened	Fishes	2	Considered	N/A	N/A
*Thomomys mazama glacialis*	Roy Prairie pocket gopher	Pacific	Threatened	Mammals	2	Considered	N/A	N/A
*Thomomys mazama yelmensis*	Yelm pocket gopher	Pacific	Threatened	Mammals	1	Considered	N/A	N/A
*Thomomys mazama pugetensis*	Olympia pocket gopher	Pacific	Threatened	Mammals	2	Considered	N/A	N/A

Assisted migration or captive outcrossing for purposes of genetic rescue were implemented in 3 species. “Genetic rescue” was considered in 8 additional species.

### Variation in GR index across taxonomic groups and regions

**Fig. 2.**
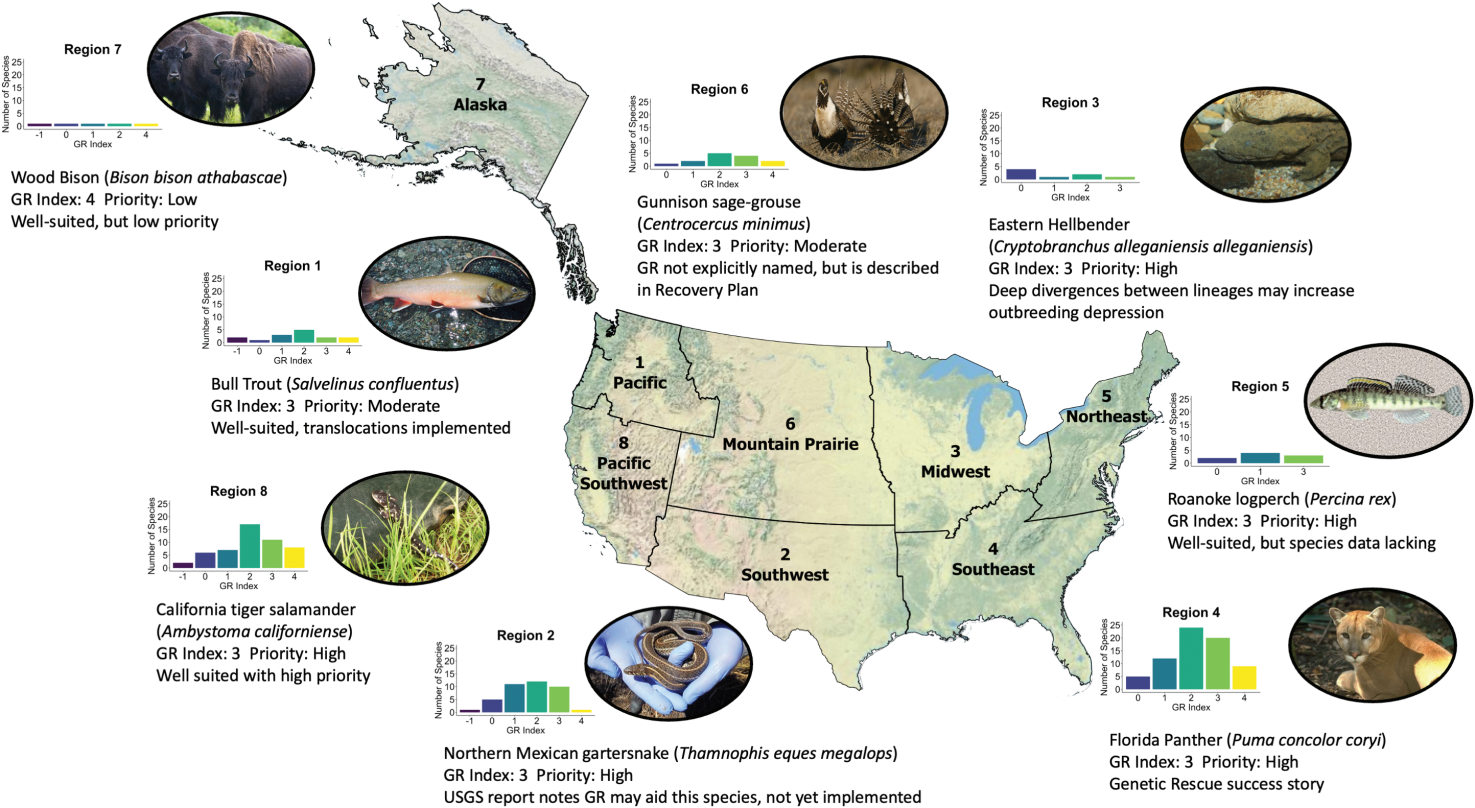
Map of contiguous United States and Alaska divided into Lead Regions according to the U.S. Fish & Wildlife Service. The distribution of GR index across species in each Lead Region and a featured vertebrate from each Lead Region are shown. Hellbender photograph taken by Brian Gratwicke (https://creativecommons.org/licenses/by/2.0/deed.en).

We did not find support for differences in GR indices across taxonomic groups, geographic regions in the United States, number of populations remaining, age at maturity, or listing status, suggesting there is not a particular “type” of species that stands to benefit from *genetic rescue*. While the number of listed species or subspecies in each taxonomic group varied from 24 (reptiles) to 95 (fishes), all groups exhibited similar distributions of GR indices (*H* = 7.66, *P* = 0.10), with most species assigned a GR index between 1 and 3 (Fig. 1B). The number of listed species per Lead Region ranged from 5 species listed in Alaska (Lead Region 7) to 71 species listed in the Southeast (Lead Region 4), but distributions of GR indices across Lead Regions did not meet the significance threshold (*H* = 13.55, *P* = 0.06; Fig. 2). Rather, differences in sample size per Lead Region seemed to obscure differences in GR index distribution. For example, 44% of species in the Midwest had a GR index = 0, which is proportionally more zeroes than other regions, but this is based on a total of only 9 listed species in the Midwest. Similarly, GR index distributions did not differ between species with fast or slow life histories (*W* = 3,151, *P* = 0.10; Supplementary Fig. S1) or between threatened versus endangered species (*W* = 6,658, *P* = 0.21). Finally, we detected no significant differences in the distribution of GR indices across species with different numbers of populations remaining when treating as either a categorical variable (*H* = 7.39, *P* = 0.49) or as an ordinal variable (Kendall’s *τ* = 0.01, *P* = 0.86). All groups and regions contained species that would be considered good candidates for *assisted migration*, indicating potential widespread benefits of this conservation strategy.

We found that all 145 species with GR indices ≥2 had at least 1 “small population concern” noted in the recovery documentation or primary literature (i.e. Q1 to Q3), indicating some uniformity in what makes a strong candidate for *genetic rescue* according to our criteria. On the other hand, there were no consistent trends underlying poor candidates (GR index <2). For example, the Endangered Indiana Bat (GR index = 0) was missing very little information, but concerns about both disease and cave site fidelity led to a low GR index for this species. In contrast, many species received low indices due to missing information. Most species were missing some information; we evaluated only 3 species with complete information across all survey questions. As expected, we found a negative relationship between number of survey questions answered “NA” and GR index (Kendall’s *τ* = −0.38, *P* << 0.01). That is, species with more missing information tended to have lower GR indices. Interestingly, missing information did not preclude species from having a high GR index. Of the 145 species with indices ≥2, 87% had more than 1 question with missing information. A lack of information should therefore not prevent evaluation of the feasibility of *assisted migration*, especially given that such an evaluation would likely fill in valuable natural history knowledge and biology for the species. The questions with the most missing information were about characterizing behavioral and/or adaptive variation among populations (Supplementary Fig. S2).

### Is *assisted migration* for *genetic rescue* underused?

**Fig. 3.**
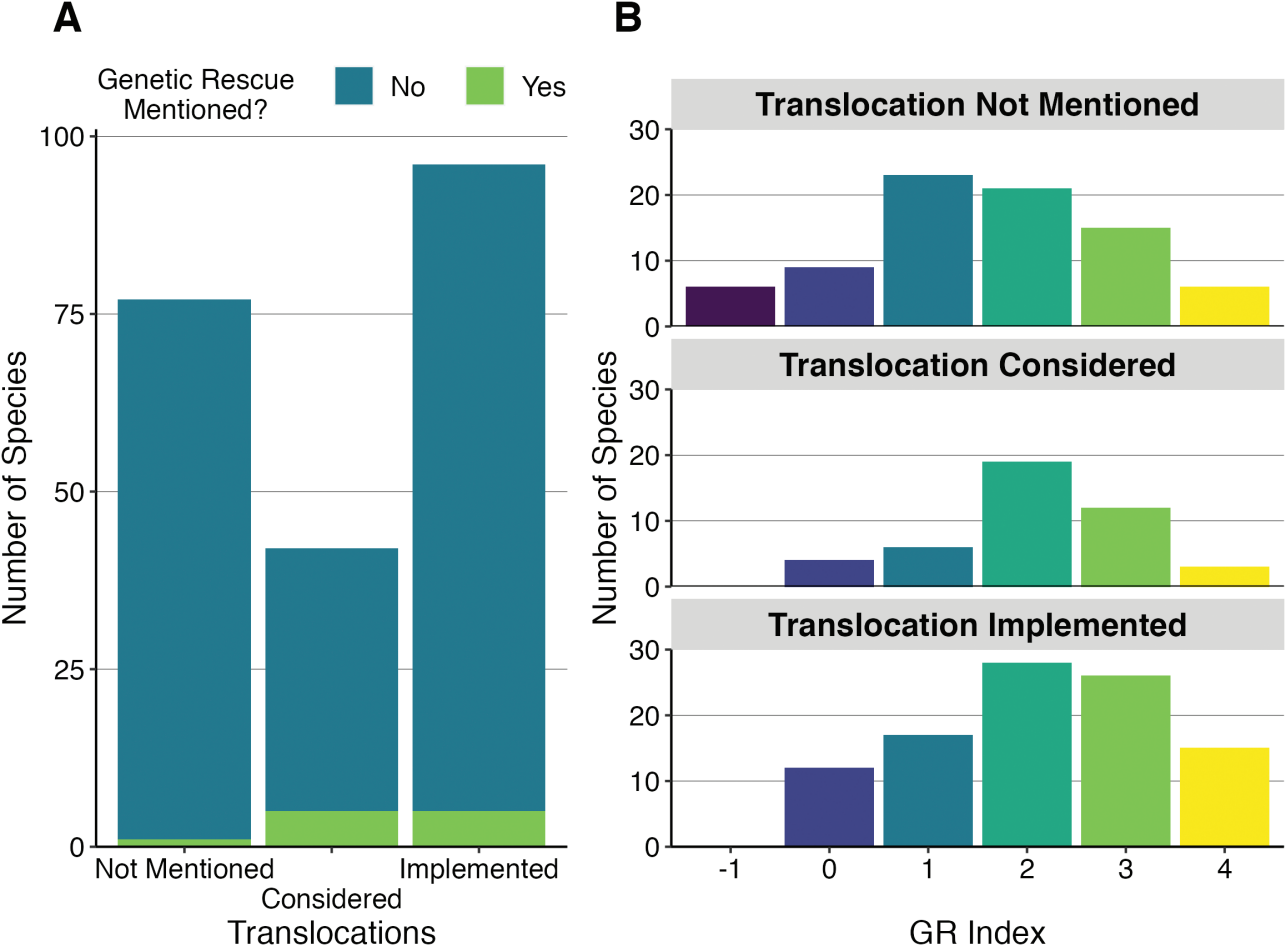
Distribution of translocation status and GR index across evaluated species. A) Stacked bar graph with translocation status colored by whether genetic rescue was mentioned in recovery documents. B) Distribution of GR index according to translocation status.

To evaluate whether *assisted migration* for *genetic rescue* is underused among candidate species (i.e. GR index ≥2), we explored patterns of translocation-related management actions in these species (i.e. any form of human-assisted movement of animals from one location to another, regardless of intention). Translocations were surprisingly frequent across taxonomic groups. Specifically, we found that translocations had been implemented in 44% of species and considered in an additional 20% of species. After accounting for variation in number of species within each taxonomic group, we found that translocation status (implemented, considered, or “not mentioned”) was significantly associated with taxonomic group (*P* = 0.03); translocations were implemented more frequently in fishes and mammals. However, despite nearly half of all evaluated species having translocations implemented, few of these management actions were carried out for the purpose of *genetic rescue*. Only 5 out of 98 species for which translocations were performed had explicit mention of *genetic rescue* in the recovery documentation, and we found only 3 instances of actual implementation as the other 2 were relocations into unoccupied habitat (Fig. 3A). For freshwater fishes, the group with the most translocations, the most common strategy was the reintroduction of captively propagated individuals into previously occupied (now, unoccupied) sites (George *et al.* 2009b). For example, hatchery-reared bluemask darters have been released into 3 unoccupied sites on the Calfkiller River in Tennessee (Taylor *et al.* 2021). In contrast, we found very few examples of translocations into an existing population, and those few cases seemed to be for the purpose of supplementing the population rather than restoring *gene flow*. In the case of the Little Kern golden trout, stocking trout reared in captivity was carried out for the purpose of increasing population size, yet this led to swamping of wild genotypes and an overall reduction in genetic variation (Lusardi *et al.* 2015). We also found that, while translocations were frequent across the species surveyed, translocations typically occur when only a few wild populations remain. The distribution in “number of populations remaining” varied significantly across translocation categories (*H* = 11.089, *P* < 0.01; Supplementary Fig. S3), such that species for which translocations were implemented tended to have fewer remaining populations than species for which translocations had not been performed or considered (Dunn’s *z* = 2.86, *P* < 0.01 and Dunn’s *z* = −2.62, *P* = 0.01, respectively).

Even though translocations were not typically implemented for the purpose of *genetic rescue*, we did find that a species’ translocation status (implemented, considered, or “not mentioned”) was positively associated with median GR index (*H* = 8.40, *P* = 0.02; Fig. 3B). That is, species with a record of translocations had higher GR indices more frequently than those for which translocations were not mentioned (Dunn’s *z* = 2.77, *P* < 0.01). This is presumably because species with a history of translocations were already determined to be well-suited to human-assisted movement. While previous translocations were not typically carried out for the purpose of *genetic rescue*, it is encouraging that the logistics and inherent barriers to such an intervention have already been overcome for many species and scenarios. Management actions that include translocations are inherently complex (Taylor *et al.* 2017; Berger-Tal *et al.* 2020), but specific protocols and best practices for operationalizing translocations (whether for *genetic rescue* or not) continue to be developed across diverse species (George *et al.* 2009a; Fischer *et al.* 2022).

Assessing the relationship between GR index and Recovery Priority category allowed us to investigate whether the priority ranking system for endangered species is compatible with implementing *assisted migration* for *genetic rescue* in species for which it is likely to be successful. Listed species are assigned *Recovery Priority number*s to indicate where conservation efforts and funding are best directed. Despite potential for high priority rankings, species facing greater risk of extinction may be unsuitable for *genetic rescue* if, for instance, there are no source populations available for translocation. However, we found that GR index and Recovery Priority category were statistically independent (Kendall’s *τ* = 0.02, *P* = 0.65), and GR indices appeared to be similarly distributed across priority categories. Although “Recovery Potential” may correlate with factors assessed in our GR index (such as options for reconnecting populations) the definition of Recovery Potential is broad, and the Priority Number also addresses taxonomic uniqueness. Thus, there is not a direct correlation between our index and the Priority Number. Encouragingly, several high priority species are also good candidates for *assisted migration* and *genetic rescue*. These higher priority species have access to more resources than lower ranked species; thus, carrying out *assisted migration* may be financially feasible. On the other hand, several moderate priority species also scored high on the GR index. Explicitly considering suitability for *genetic rescue* in assessing Recovery Potential may open additional opportunities to implement *assisted migration* with the purpose of *genetic rescue* in listed species. If resources could be found to support *genetic rescue* management actions, these steps may help these lower priority species from proceeding further into the extinction vortex.

### Caveats

We caution against a prescriptive interpretation of the GR indices in this study and note several caveats to our approach. Our scoring system is simplistic in assigning points equally and additively across categories (e.g. +1 for concerns about small population sizes, −1 for concerns about disease). In reality, the cost–benefit analysis will be more nuanced and case specific. For example, disease transmission was a concern associated with panther translocations from Texas to Florida in the 1990s, but the risks associated with sustained inbreeding depression and depletion of genetic variation were determined to be much more detrimental to the population (Seal and Lacy 1989; Roelke *et al.* 1993). However, there may be other cases where disease concerns outweigh potential benefits of restoring *gene flow*, such as concerns about fungal pathogens in many amphibians (Scheele *et al.* 2021). In addition, the small population problems contributing to the extinction vortex likely operate multiplicatively in wild populations, as opposed to the additive way these factors were considered in our scoring system. For example, strong genetic drift and inbreeding will decrease genetic variation across the genome, which may in turn reduce disease resistance or other stress responses (Bijlsma and Loeschcke 2011). If such a scoring system were to be implemented to determine suitability for *assisted migration* in practice, we would recommend incorporating these potential interactions and weighting scores based on informative and context specific prior knowledge for the given species.

While our evaluation focused on federally listed vertebrate species in the United States, we also recognize that there are many other species throughout the world and in other taxonomic groups that would benefit from *assisted migration* for *genetic rescue*, especially in plants and arthropods where many empirical examples of *genetic rescue* have been demonstrated (Willi *et al.* 2007; Pickup *et al.* 2012; Hufbauer *et al.* 2015). Finally, we emphasize that *assisted migration* for *genetic rescue* should only be 1 aspect of a comprehensive species recovery strategy. *Genetic rescue* is most effective, and likely only possible, when there is available habitat for population expansion. While overwhelming evidence suggests changes to genetic management of fragmented populations are needed, the acquisition, restoration, and management of high-quality habitat should remain a top priority.

### Potential barriers and paths forward for increased use of *assisted migration* and *genetic rescue*

Our evaluation highlighted some likely barriers to implementation of *assisted migration* for *genetic rescue*. Research on *genetic rescue* has drastically increased in the last decade, and the general species traits underlying both successful rescue and *outbreeding depression* risk are better understood. Nonetheless, we find that *genetic rescue* is rarely mentioned explicitly in species recovery plans or 5-year reviews. Although 197 out of 222 recovery plans or reviews mentioned small populations concerns, only 11 explicitly mentioned *genetic rescue*. A central purpose of these documents is to describe and prioritize site-specific management actions necessary to achieve recovery (Hoekstra *et al.* 2002). Thus, there remains a gap between the primarily academic conservation and population geneticists calling for the paradigm shift and the management actions that are currently prioritized and implemented in Recovery Plans. This points to a strong need for increased communication among the *genetic rescue* research community and the state and federal biologists, managers, and policy makers who are writing and implementing recovery plans.

As discussed above, prioritization of *assisted migration* for *genetic rescue* in species Recovery Plans could improve species outcomes, and is consistent with recovery goals and species risk assessments. However, realizing the potential benefits of *genetic rescue* for listed species will require increased communication between all stakeholders. In assessing species for listing under the ESA, the USFWS considers the “three R’s”: *Redundancy*, *Resiliency*, and *Representation*. “*Redundancy*” is the species’ capacity to “withstand catastrophic events by spreading risk among multiple populations, or a large area”; “*Resiliency*” refers to species’ capability to “withstand stochastic disturbance” and is “positively related to population size and growth rate, and may be influenced by connectivity among populations”; lastly, “*Representation*” refers to the “ability of a species to adapt to changing environmental conditions over time as characterized by the breadth of genetic and environmental diversity within and among populations” (Smith *et al.* 2018). *Assisted migration* with the goal of restoring *gene flow* between populations can introduce new genetic variation and increase adaptive capacity (*Representation*), as well as increase population sizes and connectivity between populations (*Resiliency*). To date, most translocation and *genetic rescue* efforts are undertaken as last-ditch efforts to save imminently endangered species. Earlier consideration of *assisted migration* for *genetic rescue* could allow for more options for translocation (i.e. between localities that are more environmentally similar) and lead to more successful species recovery.

Rangewide population genomic information provides a clear first step in evaluating whether a species would benefit from *assisted migration*. We were surprised to learn that ninety percent of surveyed species in this study had some level of population genetic data reported in the primary literature or recovery documentation. However, of these, most genetic datasets were based on a small number of mitochondrial (mtDNA) or nuclear genes (77% of studies), microsatellites (74%) or both (57%). Relatively fewer species had larger SNP-based genomic datasets attributed to them (23%), but this is not surprising given the recency of applying these methods to nonmodel organisms. Traditional population genetic studies based on mtDNA/nuclear markers and microsatellites remain informative about neutral processes that affect the whole genome (i.e. relationships among populations, population structure, admixture, effective population size, etc.). However, higher-resolution genomic datasets provide far more information for designing and implementing *assisted migration* for *genetic rescue* (Allendorf *et al.* 2010; Fitzpatrick and Funk 2019; Forester and Lama 2022). For example, in the planning stages of an *assisted migration* strategy, genomic data can help inform managers about which populations would benefit most from new genetic variation (i.e. high levels of inbreeding and low genetic variation) as well as ideal donor populations (i.e. low inbreeding, high genetic variation, low differentiation at adaptive loci, low levels of structural variation relative to recipient, etc.). Genomic data can also be crucial for monitoring the outcomes of *assisted migration* and *genetic rescue* (Miller *et al.* 2012; Fitzpatrick *et al.* 2020). For instance, genomic monitoring data collected after *assisted migration* could be used to confirm that translocated individuals are breeding with local residents, to track changes in genetic variation over time, and to fine-tune future management efforts. Relative to species with small marker-based datasets (i.e. mtDNA or microsatellites), we found that more species with genomic data had translocations reported as implemented or considered in their recovery documentation, although still only 16% of these explicitly mention *genetic rescue* in the recovery documentation. Thus, while it was encouraging that so many species on our survey list had at least some knowledge about population genetic patterns, the collection of higher-resolution genomic datasets for more species should continue to be a high priority for management, as these datasets are directly relevant to informing the potential for *genetic rescue*.

## Conclusions

We have formally established that *assisted migration* for *genetic rescue* is an underused strategy for the imperiled vertebrates of the United States, corroborating previous calls for increased consideration of this strategy in conservation practice. Reflective of the widespread problem of habitat fragmentation and population isolation, species with high GR indices were found across all taxonomic groups and geographic regions. There seems to be high awareness of “small population problems” within species’ recovery documentation, and widespread adoption of genetic tools to help characterize such problems, though highly informative genomic datasets are still uncommon. Yet, evaluation of suitability for *genetic rescue* tends to be absent from recovery literature. Incorporating such evaluations is an important first step toward wider adoption given that US FWS Species Status Assessments and recovery plans serve as the primary road maps for species recovery.

## Glossary

**assisted migration:** the intentional translocation of individuals within or outside the natural range of a species (Aitken and Whitlock 2013).

**Distinct Population Segment (DPS):** a vertebrate population or group of populations i.e. discrete from the other populations of the species and significant in relation to the entire species (USFWS-NMFS 1996).

**gene flow:** movement of genetic material between populations caused by migration and subsequent reproduction.

**genetic load:** the presence of deleterious genetic variation within a population or individual.

**genetic rescue:** an increase in population fitness (best measured by population growth rate) due to gene flow (Whiteley *et al.* 2015).

**genetic rescue suitability index (GR index):** index developed in this study to determine a species’ suitability for a management strategy of assisted migration with the purpose of genetic rescue.

**outbreeding depression:** negative fitness consequences caused by outcrossing or gene flow.

**Recovery Priority Number:** Ranking system used by the Endangered Species Act to assign relative priorities for conservation planning and action.

**Redundancy:** the capacity of a species to withstand catastrophic events, measured by the number of populations, their *resiliency*, and their distribution and connectivity.

**Resiliency:** the capacity of populations to withstand stochastic events, measured by the size and growth rate of each population.

**Representation:** the capacity of a species to adapt to changing environmental conditions measured by the breadth of genetic or environmental diversity within and among populations of a species.

**Species Status Assessments (SSA):** biological risk assessment to characterize a species’ ability to sustain populations in the wild over time based on the best scientific understanding of current and future abundance and distribution within the species’ ecological settings.

**US FWS recovery plan:** nonregulatory document that describes and justifies the research and site-specific management actions necessary to support recovery of a federally listed species or DPS, often developed with federal, state, tribal, local governmental, nongovernmental, and other interested parties (United States ESA 1983).

**5-year status reviews:** a periodic review of the status of species listed under the Endangered Species Act of 1973 i.e. conducted at least once every 5 yr, typically to assess each threatened and endangered species to determine whether its status has changed since the time of its listing.

## Supplementary Material

esad002_suppl_Supplementary_Material

## Data Availability

The resulting information from the species list and scoring, R code used for the survey questions can be found at https://doi.org/10.5061/dryad.7h44j0zz9.

## References

[CIT0001] Aitken SN , Whitlock MC. Assisted gene flow to facilitate local adaptation to climate change. Annu Rev Ecol. 2013;44:367–388.

[CIT0002] Allendorf FW , Hohenlohe PA, Luikart G. Genomics and the future of conservation genetics. Nat Rev Genet. 2010;11:697–709. 20847747 10.1038/nrg2844

[CIT0003] Bell DA , Robinson ZL, Funk WC, Fitzpatrick SW, Allendorf FW, Tallmon DA, Whiteley AR. The exciting potential and remaining uncertainties of genetic rescue. Trends Ecol Evol. 2019;34:1070–1079. 31296345 10.1016/j.tree.2019.06.006

[CIT0004] Berger-Tal O , Blumstein DT, Swaisgood RR. Conservation translocations: a review of common difficulties and promising directions. Anim Conserv. 2020;23:121–131.

[CIT0005] Bertorelle G , Raffini F, Bosse M, Bortoluzzi C, Iannucci A, Trucchi E, Morales E, Oosterhout C. Genetic load: genomic estimates and applications in non-model animals. Nat Rev Genet. 2022;23:1–12. 35136196 10.1038/s41576-022-00448-x

[CIT0006] Bijlsma R , Bundgaard J, Boerema AC. Does inbreeding affect the extinction risk of small populations? Predictions from *Drosophila*. J Evol Biol. 2000;13:502–514.

[CIT0007] Bijlsma R , Loeschcke V. Genetic erosion impedes adaptive responses to stressful environments. Evol Appl. 2011;5:117–129. 25568035 10.1111/j.1752-4571.2011.00214.x PMC3353342

[CIT0008] Bozzuto C , Biebach I, Muff S, Ives AR, Keller LF. Inbreeding reduces long-term growth of Alpine ibex populations. Nat Ecol Evol. 2019;3:1359–1364. 31477848 10.1038/s41559-019-0968-1

[CIT0009] Bürger R , Lynch M. Evolution and extinction in a changing environment: a quantitative-genetic analysis. Evolution. 1995;49:151–163. 28593664 10.1111/j.1558-5646.1995.tb05967.x

[CIT0010] Ceballos G , Ehrlich PR, Dirzo R. Biological annihilation via the ongoing sixth mass extinction signaled by vertebrate population losses and declines. Proc Natl Acad Sci USA. 2017;114:E6089–E6096. 28696295 10.1073/pnas.1704949114 PMC5544311

[CIT0011] Comber SCL , Smith C. Polyploidy in fishes: patterns and processes. Biol J Linn Soc. 2004;82:431–442.

[CIT0012] Cunningham AA. Disease risks of wildlife translocations. Conserv Biol. 1996;10:349–353.

[CIT0013] Didham RK , Kapos V, Ewers RM. Rethinking the conceptual foundations of habitat fragmentation research. Oikos. 2012;121:161–170.

[CIT0014] Dinno A. dunn.test: Dunn’s test of multiple comparisons using rank sums. 2017.

[CIT0015] Edmands S. Between a rock and a hard place: evaluating the relative risks of inbreeding and outbreeding for conservation and management. Mol Ecol. 2006;16:463–475. 10.1111/j.1365-294X.2006.03148.x 17257106

[CIT0016] Elias BA , Shipley LA, McCusker S, Sayler RD, Johnson TR. Effects of genetic management on reproduction, growth, and survival in captive endangered pygmy rabbits (*Brachylagus idahoensis*). J Mammal. 2013;94:1282–1292. 32287380 10.1644/12-MAMM-A-224.1 PMC7108654

[CIT0017] Fischer JH , Wittmer HU, Kenup CF, Parker KA, Cole R, Debski I, Taylor GA, Ewen JG, Armstrong D. Predicting harvest impact and establishment success when translocating highly mobile and endangered species. J Appl Ecol. 2022;59:2071–2083.

[CIT0018] Fitzpatrick SW , Bradburd GS, Kremer CT, Salerno PE, Angeloni LM, Funk WC. Genomic and fitness consequences of genetic rescue in wild populations. Curr Biol. 2020;30:517–522.e5. 31902732 10.1016/j.cub.2019.11.062

[CIT0019] Fitzpatrick SW , Funk WC. Genomics for genetic rescue. In: Hohenlohe PA, editor. Population genomics: wildlife. Switzerland: Springer; 2019.

[CIT0020] Fitzpatrick SW , Gerberich JC, Angeloni LM, Bailey LL, Broder ED, Dowdall JT, Handelsman CA, López-Sepulcre A, Reznick DN, Ghalambor CK, Funk WC. Gene flow from an adaptively divergent source causes rescue through genetic and demographic factors in two wild populations of Trinidadian guppies. Evol Appl. 2016;9:879–891. 27468306 10.1111/eva.12356 PMC4947150

[CIT0021] Forester BR , Lama TM. The role of genomics in the future of ESA decision-making. In: Baier LE, Organ J, editors. The Codex of the Endangered Species Act: the next fifty years. Vol. II. 2022.

[CIT0022] Frankham R. Genetics and extinction. Biol Conserv. 2005;126:131–140.

[CIT0023] Frankham R. Genetic rescue of small inbred populations: meta-analysis reveals large and consistent benefits of gene flow. Mol Ecol. 2015;24:2610–2618. 25740414 10.1111/mec.13139

[CIT0024] Frankham R. Genetic rescue benefits persist to at least the F3 generation, based on a meta-analysis. Biol Conserv. 2016;195:33–36.

[CIT0025] Frankham R , Ballou JD, Eldridge MDB, Lacy RC, Ralls K, Dudash MR, Fenster Charles B. Predicting the probability of outbreeding depression. Conserv Biol. 2011;25:465–475. 21486369 10.1111/j.1523-1739.2011.01662.x

[CIT0026] Frankham R , Ballou JD, Ralls K, Eldridge M, Dudash MR, Fenster CB, Sunnucks P. Genetic management of fragmented animal and plant populations. Oxford, UK: Oxford University Press; 2017.

[CIT0027] Frankham R , Ballou JD, Ralls K, Eldridge M, Dudash MR, Fenster CB, Lacy RC, Sunnucks P. A practical guide for genetic management of fragmented animal and plant populations. Oxford University Press, 2019.

[CIT0028] Frankham R , Lees K, Montgomery ME, England PR, Lowe EH, Brisco DA. Do population size bottlenecks reduce evolutionary potential? Anim Conserv. 1999;2:255–260.

[CIT0029] Gabriel W , Bürger R. Survival of small populations under demographic stochasticity. Theor Popul Biol. 1992;41:44–71. 1604427 10.1016/0040-5809(92)90049-y

[CIT0030] George AL , Kuhajda BR, Williams JD, Cantrell MA, Rakes PL, Shute JR. Guidelines for propagation and translocation for freshwater fish conservation. Fisheries. 2009a;34:529–545.

[CIT0031] George AL , Neely DA, Mayden RL. Conservation genetics of an imperiled riverine fish from Eastern North America, the Blotchside Logperch, *Percina burtoni* (Teleostei: Percidae). Copeia. 2009b;2006:585–594.

[CIT0032] Hasselgren M , Angerbjörn A, Eide NE, Erlandsson R, Flagstad O, Landa A, Wallén J, Norén K. Genetic rescue in an inbred Arctic fox (*Vulpes lagopus*) population. Proc R Soc B Biol Sci. 2018;285:20172814. 10.1098/rspb.2017.2814 PMC5897638 29593110

[CIT0033] Hedrick PW , Garcia-Dorado A. Understanding inbreeding depression, purging, and genetic rescue. Trends Ecol Evol. 2016;31:940–952. 27743611 10.1016/j.tree.2016.09.005

[CIT0034] Hoekstra JM , Clark JA, Fagan WF, Boersma PD. A comprehensive review of Endangered Species Act recovery plans. Ecol Appl. 2002;12:630–640.

[CIT0035] Hoffmann AA , Miller AD, Weeks AR. Genetic mixing for population management: from genetic rescue to provenancing. Evol Appl. 2021;14:634–652. 33767740 10.1111/eva.13154 PMC7980264

[CIT0036] Hogg JT , Forbes SH, Steele BM, Luikart G. Genetic rescue of an insular population of large mammals. Proc R Soc B Biol Sci. 2006;273:1491–1499. 10.1098/rspb.2006.3477 PMC1560318 16777743

[CIT0037] Hufbauer RA , Szűcs M, Kasyon E, Youngberg C, Koontz MJ, Richards C, Tuff T, Melbourne BA. Three types of rescue can avert extinction in a changing environment. Proc Natl Acad Sci USA. 2015;112:10557–10562. 26240320 10.1073/pnas.1504732112 PMC4547288

[CIT0038] Johnson WE , Onorato DP, Roelke ME, Land ED, Cunningham M, Belden RC, McBride R, Jansen D, Lotz M, Shindle D, et al. Genetic restoration of the Florida panther. Science. 2010;329:1641–1645. 20929847 10.1126/science.1192891 PMC6993177

[CIT0039] Kardos M , Armstrong EE, Fitzpatrick SW, Hauser S, Hedrick PW, Miller JM, Tallmon DA, Funk WC. The crucial role of genome-wide genetic variation in conservation. Proc Natl Acad Sci USA. 2021;118:e2104642118. 34772759 10.1073/pnas.2104642118 PMC8640931

[CIT0040] Keller LF , Waller DM. Inbreeding effects in wild populations. Trends Ecol Evol. 2002;17:230–241.

[CIT0041] Klerks PL , Athrey GN, Leberg PL. Response to selection for increased heat tolerance in a small fish species, with the response decreased by a population bottleneck. Front Ecol Evol. 2019;7:95–10.

[CIT0042] Kronenberger JA , Funk WC, Smith JW, Fitzpatrick SW, Angeloni LM, Broder ED, Ruell EW. Testing the demographic effects of divergent immigrants on small populations of Trinidadian guppies. Anim Conserv. 2017;20:3–11.

[CIT0043] Kronenberger JA , Gerberich JC, Fitzpatrick SW, Broder ED, Angeloni LM, Funk WC. An experimental test of alternative population augmentation scenarios. Conserv Biol. 2018;32:838–848. 29349820 10.1111/cobi.13076

[CIT0044] Lande R. Risks of population extinction from demographic and environmental stochasticity and random catastrophes. Am Nat. 1993;142:911–927. 29519140 10.1086/285580

[CIT0045] Lennartsson T. Extinction thresholds and disrupted plant-pollinator interactions in fragmented plant populations. Ecology. 2002;83:3060–3072.

[CIT0046] Lusardi RA , Stephens MR, Moyle PB, McGuire CL, Hull JM. Threat evolution: negative feedbacks between management action and species recovery in threatened trout (Salmonidae). Rev Fish Biol Fisher. 2015;25:521–535.

[CIT0047] Miller JM , Poissant J, Hogg JT, Coltman DW. Genomic consequences of genetic rescue in an insular population of bighorn sheep (*Ovis canadensis*). Mol Ecol. 2012;21:1583–1596. 22257293 10.1111/j.1365-294X.2011.05427.x

[CIT0048] Nerkowski SA. A Rabbit’s Tale: genetic monitoring, genomic diversity, and habitat selection in the endangered Columbia Basin pygmy rabbit (*Brachylagus idahoensis*) [doctoral dissertation]. Idaho, US: University of Idaho; 2021.

[CIT0049] Newman D , Pilson D. Increased probability of extinction due to decreased genetic effective population size: experimental populations of *Clarkia pulchella*. Evolution. 1997;51:354–362. 28565367 10.1111/j.1558-5646.1997.tb02422.x

[CIT0050] Pedersen, TL. Patchwork: The composer of plots. R package version 1. 2020;182.

[CIT0051] Pickup M , Field DL, Rowell DM, Young AG. Source population characteristics affect heterosis following genetic rescue of fragmented plant populations. Proc R Soc B Biol Sci. 2012;280:20122058. 10.1098/rspb.2012.2058 PMC3574427 23173202

[CIT0052] Ralls K , Ballou JD, Dudash MR, Eldridge MDB, Fenster CB, Lacy RC, Sunnucks P, Frankham R. Call for a paradigm shift in the genetic management of fragmented populations. Conserv Lett. 2018;11:e12412.

[CIT0053] Ralls K , Sunnucks P, Lacy RC, Frankham R. Genetic rescue: a critique of the evidence supports maximizing genetic diversity rather than minimizing the introduction of putatively harmful genetic variation. Biol Conserv. 2020;251:108784.

[CIT0054] Robinson ZL , Bell DA, Dhendup T, Luikart G, Whiteley AR, Kardos M. Evaluating the outcomes of genetic rescue attempts. Conserv Biol. 2021;35:666–677. 32700770 10.1111/cobi.13596

[CIT0055] Robinson ZL , Coombs JA, Hudy M, Nislow KH, Letcher BH, Whiteley AR. Experimental test of genetic rescue in isolated populations of brook trout. Mol Ecol. 2017;26:4418–4433. 28664980 10.1111/mec.14225

[CIT0056] Roelke ME , Martenson JS, O’Brien SJ. The consequences of demographic reduction and genetic depletion in the endangered Florida panther. Curr Biol. 1993;3:340–350. 15335727 10.1016/0960-9822(93)90197-v

[CIT0057] Saccheri I , Kuussaari M, Kankare M, Vikman P, Fortelius W, Hanski I. Inbreeding and extinction in a butterfly metapopulation. Nature. 1998;392:491–494.

[CIT0058] Scheele BC , Hollanders M, Hoffmann EP, Newell DA, Lindenmayer DB, McFadden M, Gilbert DJ, Grogan LF. Conservation translocations for amphibian species threatened by chytrid fungus: a review, conceptual framework, and recommendations. Conserv Sci Pract. 2021;3:2578–4854.

[CIT0059] Schmidt-Lebuhn AN , Marshall DJ, Dreis B, Young AG. Genetic rescue in a plant polyploid complex: case study on the importance of genetic and trait data for conservation management. Ecol Evol. 2018;8:5153–5163. 29876089 10.1002/ece3.4039 PMC5980434

[CIT0060] Seal US , Lacy RC. Florida panther population viability analysis. Apple Valey (MN): Captive Breeding Specialists Group, Species Survival Commission, IUCN, Report to the US Fish and Wildlife Service; 1989.

[CIT0061] Service UF and W. US Fish and Wildlife Service. “Policy regarding the recognition of distinct vertebrate population segments under the Endangered Species Act.” Federal Register. 1996;61:4722.

[CIT0062] Skelly DK , Joseph LN, Possingham HP, Freidenburg LK, Farrugia TJ, Kinnison MT, Hendry AP. Evolutionary responses to climate change. Conserv Biol. 2007;21:1353–1355. 17883501 10.1111/j.1523-1739.2007.00764.x

[CIT0063] Smith D , Allan NL, McGowan CP, Szymanski J, Oetker SR, Bell HM. Development of a species status assessment process for decisions under the U.S. Endangered Species Act. J Fish Wildl Manag. 2018;9:302–320.

[CIT0064] Tallmon DA , Luikart G, Waples RS. The alluring simplicity and complex reality of genetic rescue. Trends Ecol Evol. 2004;19:489–496. 16701312 10.1016/j.tree.2004.07.003

[CIT0065] Taylor LU , Benavides E, Simmons JW, Near TJ. Genomic and phenotypic divergence informs translocation strategies for an endangered freshwater fish. Mol Ecol. 2021;30:3394–3407. 33960044 10.1111/mec.15947

[CIT0066] Taylor HR , Dussex N, van Heezik Y. Bridging the conservation genetics gap by identifying barriers to implementation for conservation practitioners. Glob Ecol Conserv. 2017;10:231–242.

[CIT0067] Team RC. R: a language and environment for statistical computing. Vienna (Austria): R Foundation for Statistical Computing; 2022.

[CIT0068] United States. The Endangered Species Act as amended by Public Law 97-304 (the Endangered Species Act amendments of 1982). Washington: U.S. G.P.O.; 1983.

[CIT0069] Urbanek S. png: Read and write PNG images. R package version 0.1-7 (2013).

[CIT0070] Vilas C , Miguel ES, Amaro R, Garcia C. Relative contribution of inbreeding depression and eroded adaptive diversity to extinction risk in small populations of shore campion. Conserv Biol. 2006;20:229–238. 16909676 10.1111/j.1523-1739.2005.00275.x

[CIT0071] Weeks AR , Heinze D, Perrin L, Stoklosa J, Hoffmann AA, van Rooyen A, Anthony van Kelly T, Mansergh I. Genetic rescue increases fitness and aids rapid recovery of an endangered marsupial population. Nat Commun. 2017;8:1071. 29057865 10.1038/s41467-017-01182-3 PMC5715156

[CIT0072] Weeks AR , Sgro CM, Young AG, Frankham R, Mitchell NJ, Miller KA, Byrne M, Coates DJ, Eldridge MDB, Sunnucks P, Breed MF, James EA, Hoffman AA. Assessing the benefits and risks of translocations in changing environments: a genetic perspective. Evol Appl. 2011;4:709–725. 22287981 10.1111/j.1752-4571.2011.00192.x PMC3265713

[CIT0073] Westemeier RL , Brawn JD, Simpson SA, Esker TL, Jansen RW, Walk JW, Kershner EL, Bouzat JL, Paige KN. Tracking the long-term decline and recovery of an isolated population. Science. 1998;282:1695–1698. 9831558 10.1126/science.282.5394.1695

[CIT0074] Whiteley AR , Fitzpatrick SW, Funk WC, Tallmon DA. Genetic rescue to the rescue. Trends Ecol Evol. 2015;30:42–49. 25435267 10.1016/j.tree.2014.10.009

[CIT0075] Wickham H. ggplot2, elegant graphics for data analysis. R. 2016. p. 241–253.

[CIT0076] Willi Y , Kleunen M, Dietrich S, Fischer M. Genetic rescue persists beyond first-generation outbreeding in small populations of a rare plant. Proc R Soc B Biol Sci. 2007;274:2357–2364. 10.1098/rspb.2007.0768 PMC2288556 17623641

[CIT0077] Willi Y , Kristensen TN, Sgrò CM, Weeks AR, Ørsted M, Hoffmann AA. Conservation genetics as a management tool: the five best-supported paradigms to assist the management of threatened species. Proc Natl Acad Sci USA. 2022;119:e2105076119. 34930821 10.1073/pnas.2105076119 PMC8740573

[CIT0078] Wright LI , Tregenza T, Hosken DJ. Inbreeding, inbreeding depression and extinction. Conserv Genet. 2008;9:833–843.

