# Supplementary material for: Genetic rescue remains underused for aiding recovery of federally listed vertebrates in the United States
Source: J Hered. 2023 Mar 28;114(4):354–66. doi: 10.1093/jhered/esad002 (PMC10287150; doi:10.1093/jhered/esad002)

Supplemental Material for:

Genetic rescue remains underused for aiding recovery of federally listed vertebrates in the United States

Sarah W. Fitzpatrick[1,2,3], Cinnamon Mittan-Moreau[1,3], Madison Miller[4], Jessica M. Judson[1]

[1] W.K. Kellogg Biological Station, Michigan State University, Hickory Corners, MI, USA

[2] Department of Integrative Biology, Michigan State University, East Lansing, MI, USA

[3] Ecology, Evolution, and Behavior Program, Michigan State University, East Lansing, MI, USA

[4] Savannah River Ecology Lab, University of Georgia, Aiken, SC, USA

Table S1: Guidelines for determining species' recovery potential Adapted from Federal Register Document 83-25716, 1983.

	High Recovery Potential	Low Recovery Potential
**Biological and Ecological limiting factors**	Well understood	Poorly understood
**Threats to species existence**	Well understood and easily managed	Poorly understood or pervasive and difficult to alleviate
**Management needed**	Intensive management not needed, or techniques well documented with high priority of success	Intensive management with uncertain probability of success, or techniques unknown or still experimental

Figure S1. Relationship between time to maturity and Genetic Rescue Suitability Index (GR Index). Age at maturity was split into two categories: species that mature in less than 4 years are included in the "Fast" category, and those that mature in 4 or more years are included in the "Slow" category.

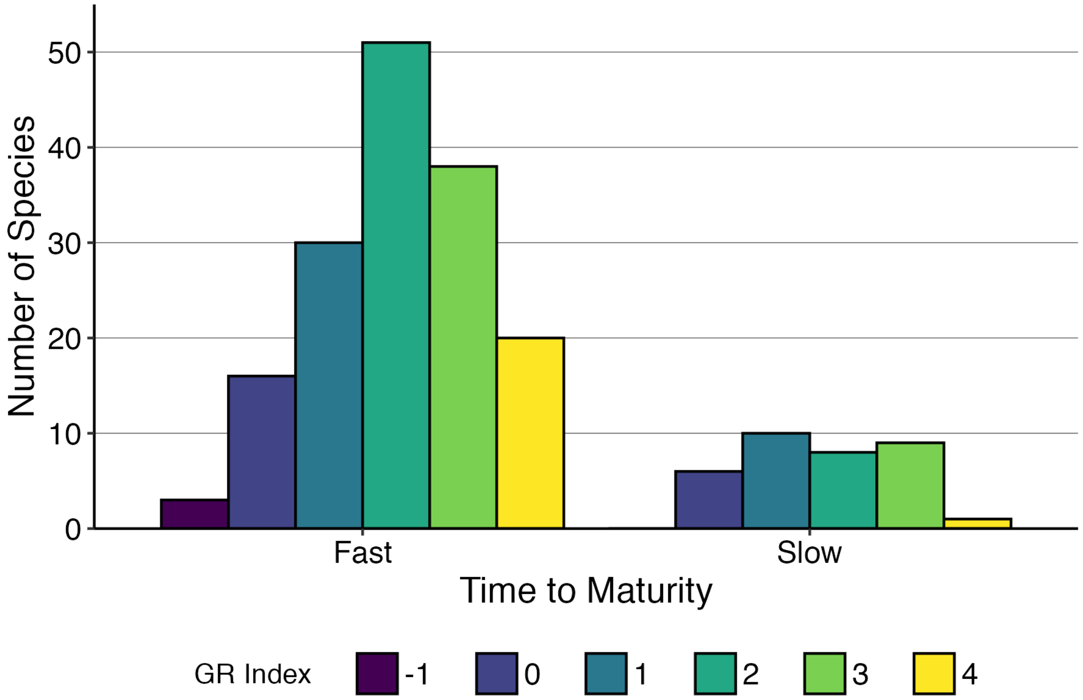

Figure S2. Percent of scores given for each question used in assigning a genetic rescue suitability index. Abbreviations for each question are on the x-axis.

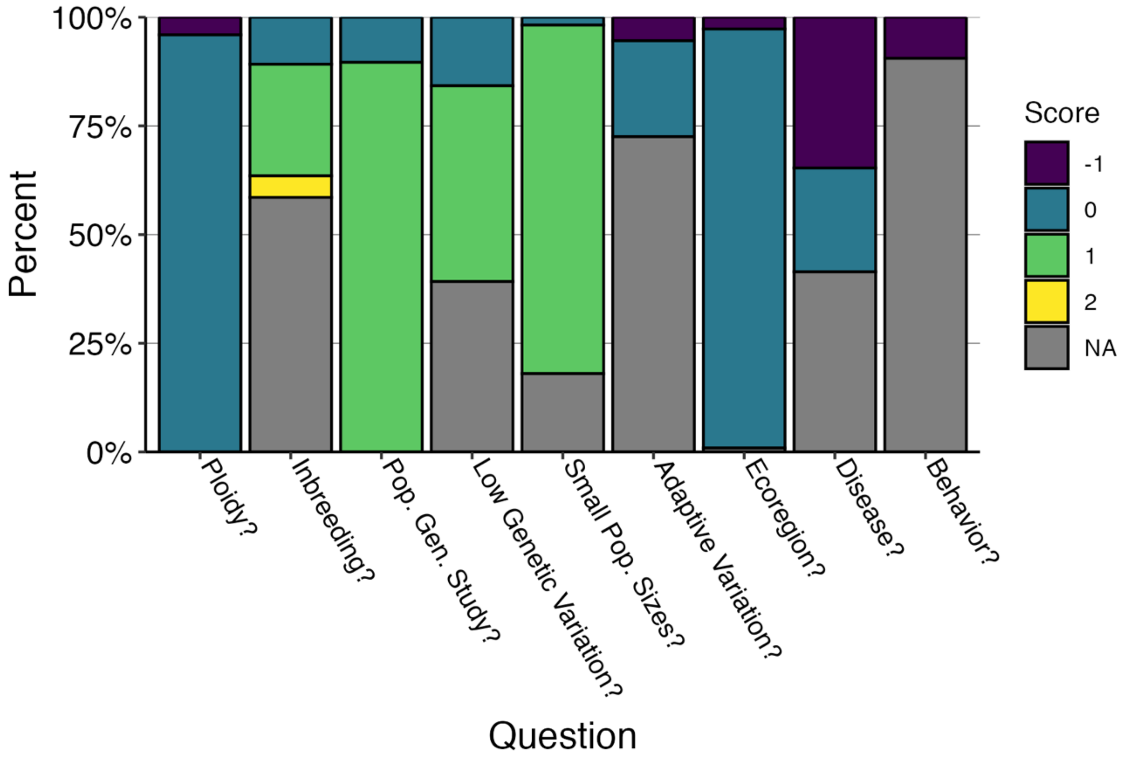

Figure S3. Proportion of species within each translocation status split by the number of populations remaining. The remaining number of populations for each species was derived from NatureServe Explorer's "Estimated Number of Element Occurrences".

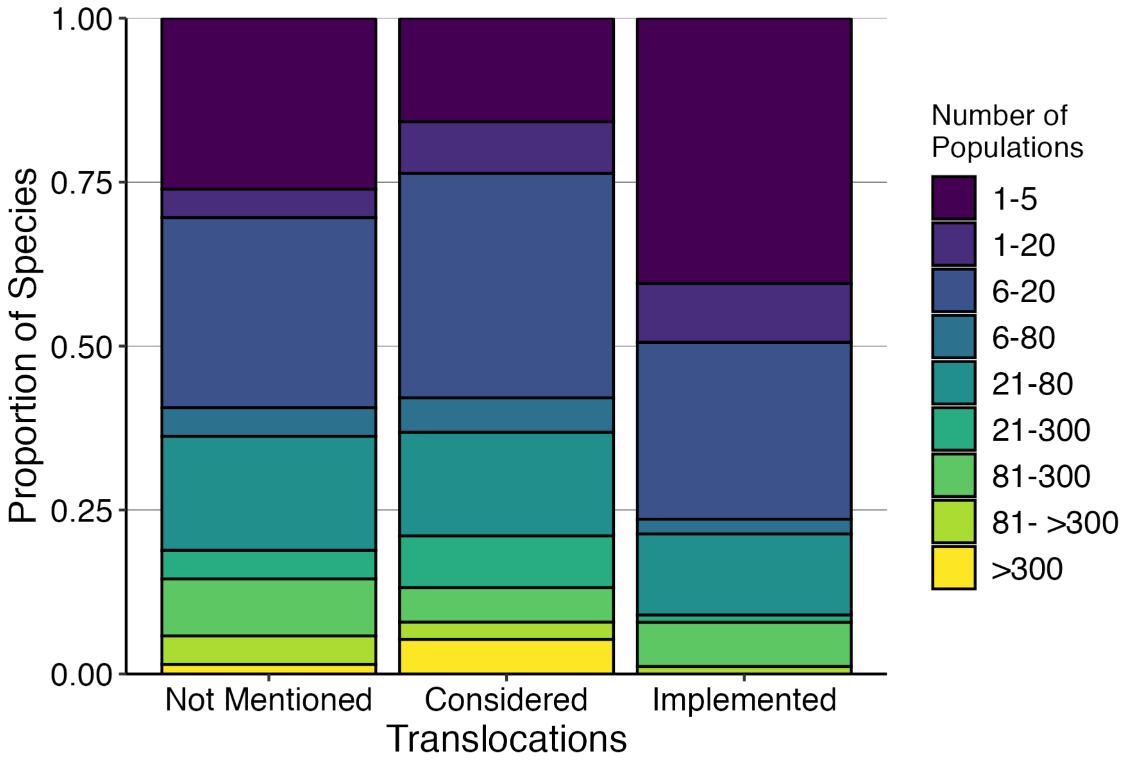

Supplement: esad002_suppl_Supplementary_Material [file esad002_suppl_supplementary_material.pdf]
